# The genome sequence of a hoverfly, *Xanthogramma pedissequum *(Harris, 1776)

**DOI:** 10.12688/wellcomeopenres.17559.1

**Published:** 2022-02-02

**Authors:** Olga Sivell, Duncan Sivell, Natural History Museum Genome Acquisition Lab, Darwin Tree of Life Barcoding collective, Wellcome Sanger Institute Tree of Life programme, Wellcome Sanger Institute Scientific Operations: DNA Pipelines collective, Tree of Life Core Informatics collective, Darwin Tree of Life Consortium

**Affiliations:** 1 Department of Life Sciences, Natural History Museum, London, UK

**Keywords:** Xanthogramma pedissequum, genome sequence, chromosomal, Diptera

## Abstract

We present a genome assembly from an individual male *Xanthogramma pedissequum *(Arthropoda; Insecta; Diptera; Syrphidae). The genome sequence is 977 megabases in span. The majority of the assembly (95.94%) is scaffolded into six chromosomal pseudomolecules, with the X and Y sex chromosomes assembled.

## Species taxonomy

Eukaryota; Metazoa; Ecdysozoa; Arthropoda; Hexapoda; Insecta; Pterygota; Neoptera; Endopterygota; Diptera; Brachycera; Muscomorpha; Syrphoidea; Syrphidae; Syrphinae; Syrphini; Xanthogramma; *Xanthogramma pedissequum* (Harris, 1776) (NCBI:txid414876).

## Background

*Xanthogramma pedissequum* is a black and yellow wasp-mimic from the family Syrphidae (Diptera), commonly known as hoverflies. *Xanthogramma pedissequum* is found in grassland habitats, woodland rides and in suburban gardens. It occurs predominantly in southern Britain with a few scattered records reaching Scotland ( Ball *et al.,* 2015; Speight, 2017). The flight period is from May to September, peaking in late June to early July ( Speight & Sarthou, 2017; Stubbs *et al.,* 2002). Adults are often found flying low among tall plants or sitting on low growing vegetation. They feed on flowers of umbellifers and yellow composites. The larvae are predators of root aphids tended by *Lasius* ants (Hymenoptera: Formicidae, *Lasius* Fabricius, 1804) ( Speight, 2017).

This species strongly resembles *Xanthogramma stackelbergi*, which was reported in Britain for the first time in 2012 ( Stubbs, 2012b)). Keys separating those two species have been provided by Speight (2010), Speight & Sarthou (2017) and Stubbs (2012a). Both species have a yellow vertical stripe on the side of the thorax and an absence of other markings in this area would indicate *X. pedissequum*, but this character is variable and some *X. pedissequum* have additional yellow markings, similar to *X. stackelbergi*. Any *Xanthogramma* specimens with 3–5 yellow markings on the side of the thorax will therefore need to be identified using other characters, many of which are also variable ( Stubbs, 2012a).

The three species within the *Xanthogramma pedissequum* group: *X. dives* (predominantly Mediterranean), *X. pedissequum* and *X. stackelbergi* cannot be separated using COI sequences. However, *X. pedissequum* can be distinguished based on ITS2 sequence ( Nedeljković *et al*., 2018).

The high-quality genome sequence described here is the first one reported for *Xanthogramma pedissequum* and has been generated as part of the Darwin Tree of Life project. It will aid research on the taxonomy, biology and ecology of the species.

## Genome sequence report

**Figure 1.**
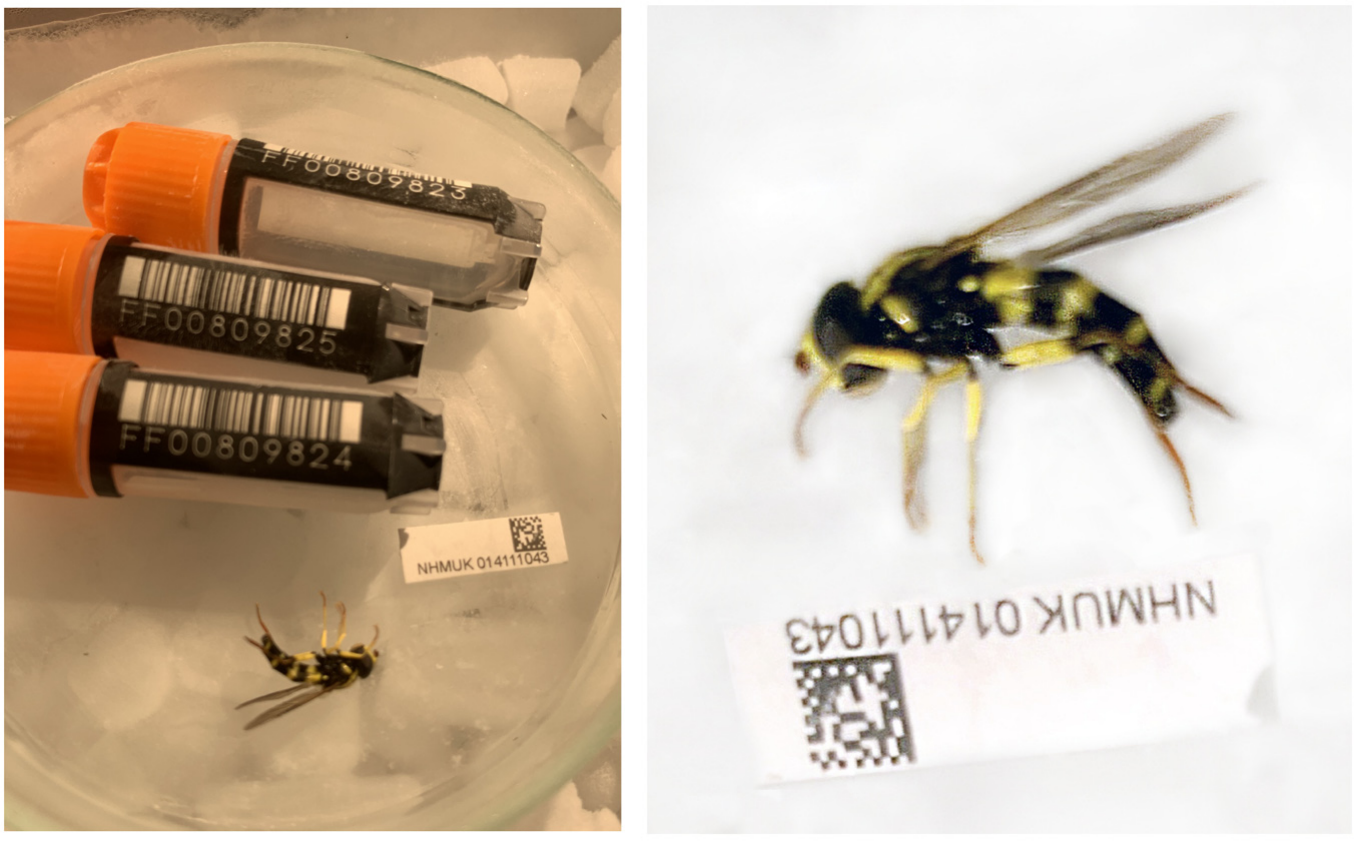
Images of the idXanPedi1 specimen taken during preservation and processing. Unfortunately, a higher-quality image of the sampled specimen is unavailable.

The genome was sequenced from a single female *X. pedissequum* ( Figure 1) collected from Wigmore Park, Luton, UK (latitude 51.88378, longitude -0.36861422). A total of 36-fold coverage in Pacific Biosciences single-molecule long reads and 57-fold coverage in 10X Genomics read clouds were generated. Primary assembly contigs were scaffolded with chromosome conformation Hi-C data. Manual assembly curation corrected 501 missing/misjoins and removed 14 haplotypic duplications, reducing the assembly size by 0.59% and the scaffold number by 23.90%, and increasing the scaffold N50 by 166.84%.

**Figure 2.**
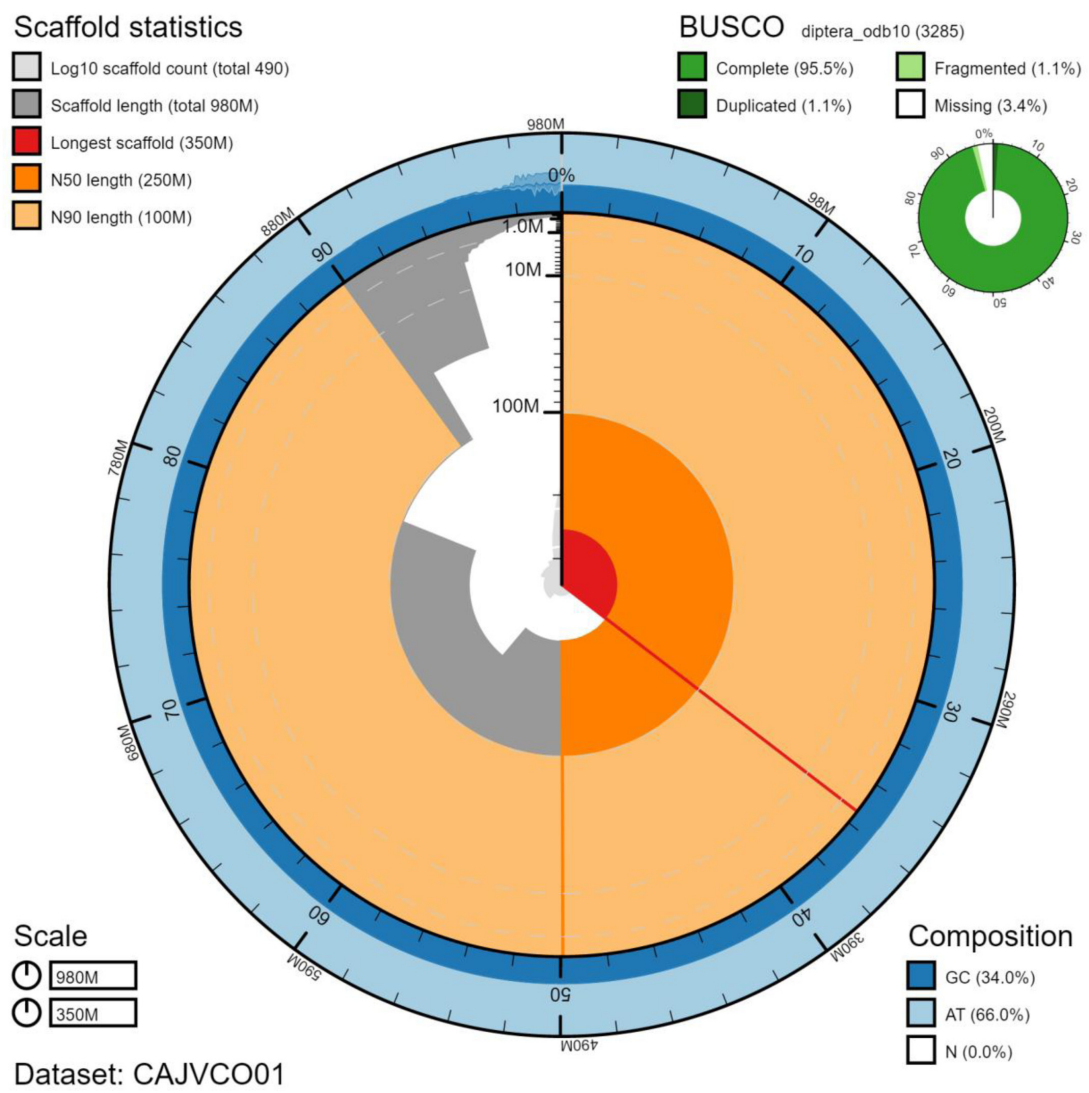
Genome assembly of *Xanthogramma pedissequum*, idXanPedi1.1: metrics. The BlobToolKit Snailplot shows N50 metrics and BUSCO gene completeness. The main plot is divided into 1,000 size-ordered bins around the circumference with each bin representing 0.1% of the 977,171,468 bp assembly. The distribution of scaffold lengths is shown in dark grey with the plot radius scaled to the longest scaffold present in the assembly (346,874,609 bp, shown in red). Orange and pale-orange arcs show the N50 and N90 scaffold lengths (248,688,513 and 101,208,079 bp), respectively. The pale grey spiral shows the cumulative scaffold count on a log scale with white scale lines showing successive orders of magnitude. The blue and pale-blue area around the outside of the plot shows the distribution of GC, AT and N percentages in the same bins as the inner plot. A summary of complete, fragmented, duplicated and missing BUSCO genes in the diptera_odb10 set is shown in the top right. An interactive version of this figure is available at https://blobtoolkit.genomehubs.org/view/idXanPedi1.1/dataset/CAJVCO01/snail.

The final assembly has a total length of 977 Mb in 484 sequence scaffolds with a scaffold N50 of 248.7 Mb ( Table 1). The majority, 95.94%, of the assembly sequence was assigned to six chromosomal-level scaffolds, representing four autosomes (numbered by sequence length), and the X and Y sex chromosome ( Figure 2– Figure 5; Table 2). Chromosome 1 contains a large, heterochromatic region of low confidence at approximately 113.44–242.17 Mb. This block consists of numerous scaffolds with high repeat content that can be localised to chromosome one but their order and orientation is unsure. Hi-C data indicates that the region 32.39–38.38Mb on Chromosome X has a strong association with scaffolds labelled as Chromosome Y and Y_unloc. This highly repetitive region is likely misassembled containing data from both X and Y that was unable to be separated due to the limitations of current technologies. The assembly has a BUSCO v5.1.2 ( Manni *et al*., 2021) completeness of 95.5% (single 94.5%, duplicated 1.1%) using the diptera_odb10 reference set. While not fully phased, the assembly deposited is of one haplotype. Contigs corresponding to the second haplotype have also been deposited.

**Table 1.** Genome data for *Xanthogramma pedissequum*, idXanPedi1.1.

*Project accession data*	
Assembly identifier	idXanPedi1.1
Species	*Xanthogramma pedissequum*
Specimen	idXanPedi1
NCBI taxonomy ID	1226616
BioProject	PRJEB45188
BioSample ID	SAMEA7521524
Isolate information	Male, thorax
*Raw data accessions*	
PacificBiosciences SEQUEL II	ERR6412036, ERR7254634
10X Genomics Illumina	ERR6054842-ERR6054845, ERR6054847-ERR6054850
Hi-C Illumina	ERR6054846
*Genome assembly*	
Assembly accession	GCA_910595825.1
*Accession of alternate haplotype*	GCA_910595765.1
Span (Mb)	977
Number of contigs	959
Contig N50 length (Mb)	7.8
Number of scaffolds	484
Scaffold N50 length (Mb)	248.7
Longest scaffold (Mb)	346.9
BUSCO * genome score	C:95.5%[S:94.5%,D:1.1%],F:1.1%,M:3.4%,n:3285

*BUSCO scores based on the diptera_odb10 BUSCO set using v5.1.2. C= complete [S= single copy, D=duplicated], F=fragmented, M=missing, n=number of orthologues in comparison. A full set of BUSCO scores is available at https://blobtoolkit.genomehubs.org/view/idXanPedi1.1/dataset/CAJVCO01/busco.

**Figure 3.**
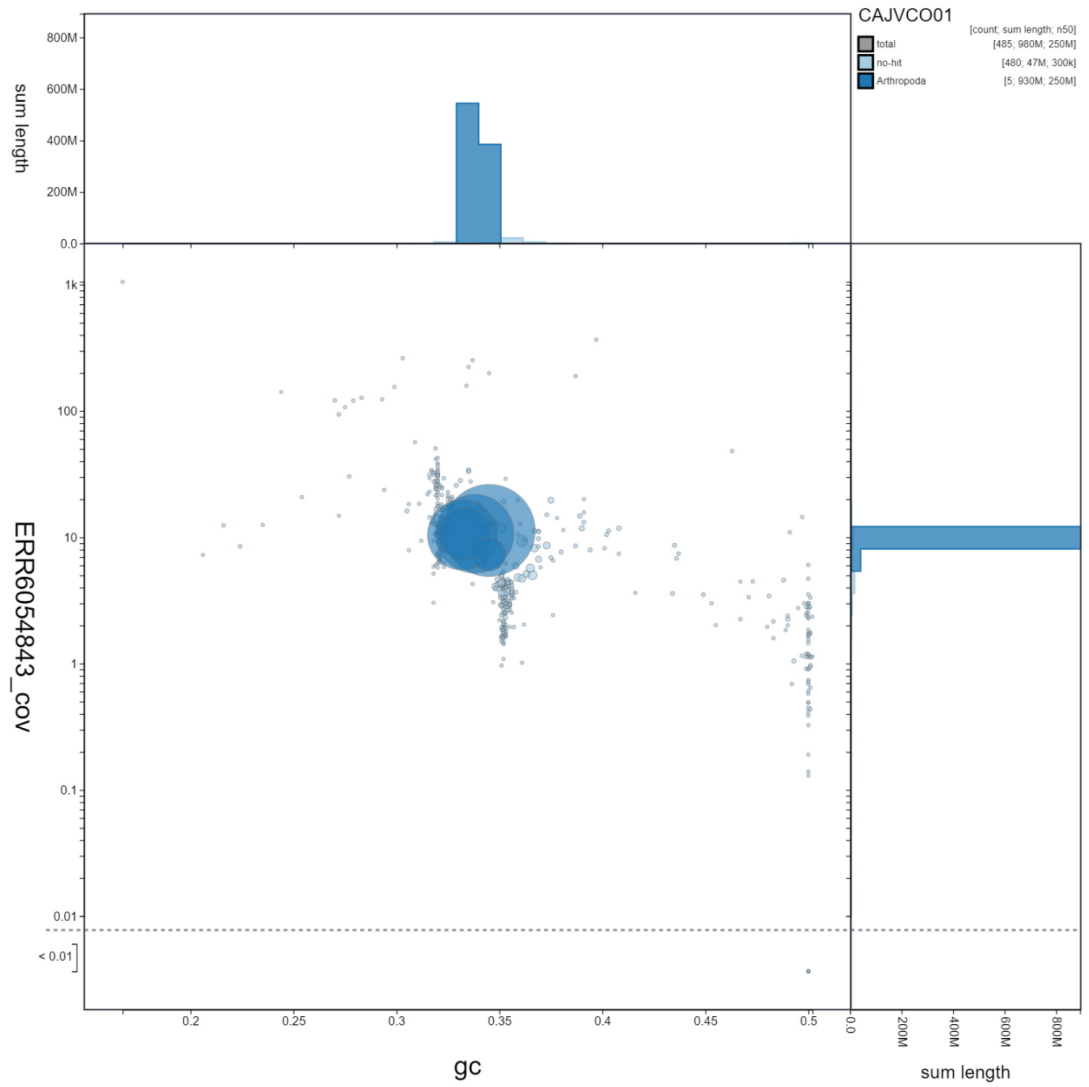
Genome assembly of *Xanthogramma pedissequum*, idXanPedi1.1: GC coverage. BlobToolKit GC-coverage plot. Scaffolds are coloured by phylum. Circles are sized in proportion to scaffold length. Histograms show the distribution of scaffold length sum along each axis. An interactive version of this figure is available at https://blobtoolkit.genomehubs.org/view/idXanPedi1.1/dataset/CAJVCO01/blob.

**Figure 4.**
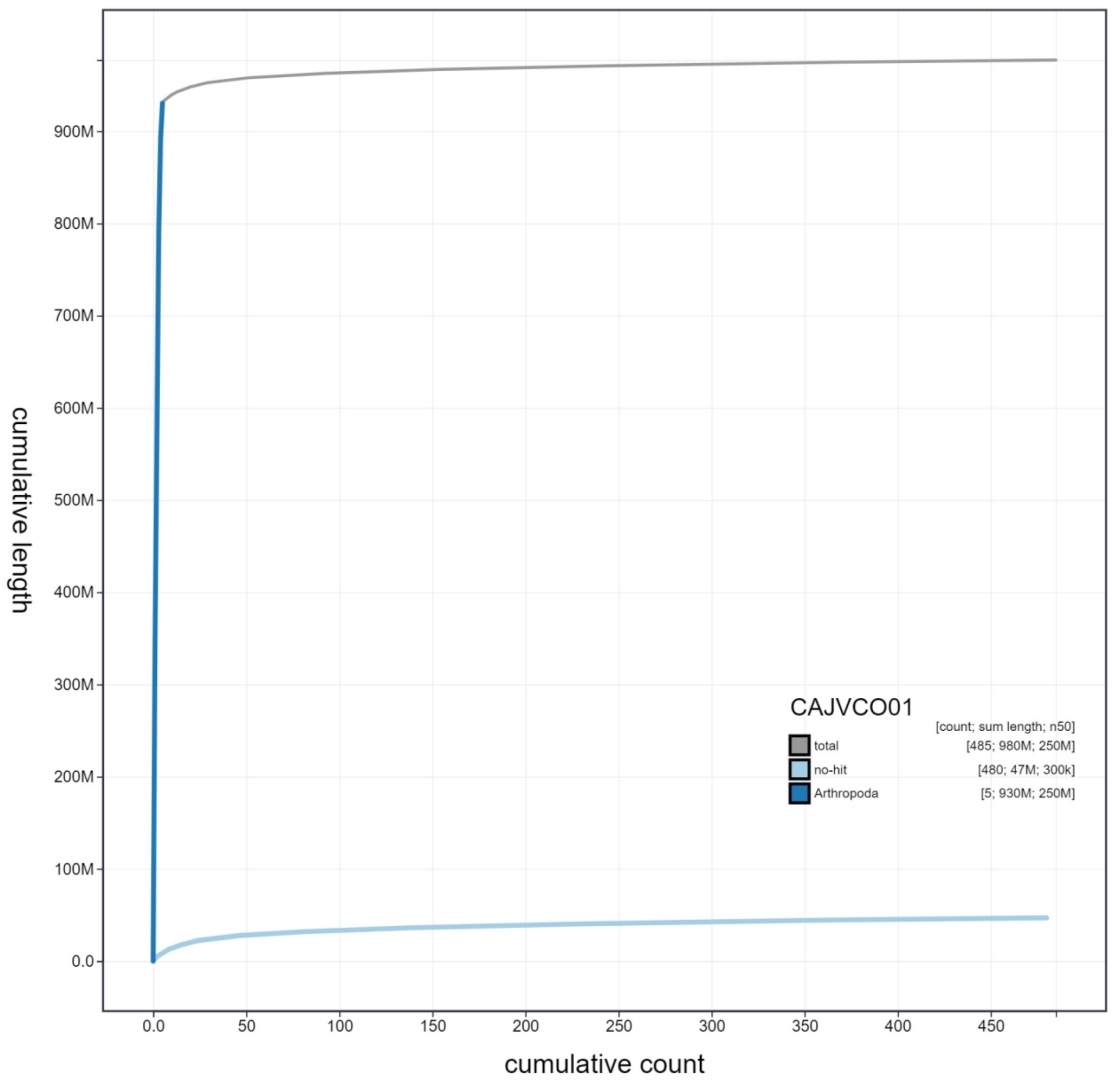
Genome assembly of *Xanthogramma pedissequum*, idXanPedi1.1: cumulative sequence. BlobToolKit cumulative sequence plot. The grey line shows cumulative length for all scaffolds. Coloured lines show cumulative lengths of scaffolds assigned to each phylum using the buscogenes taxrule. An interactive version of this figure is available at https://blobtoolkit.genomehubs.org/view/idXanPedi1.1/dataset/CAJVCO01/cumulative.

**Figure 5.**
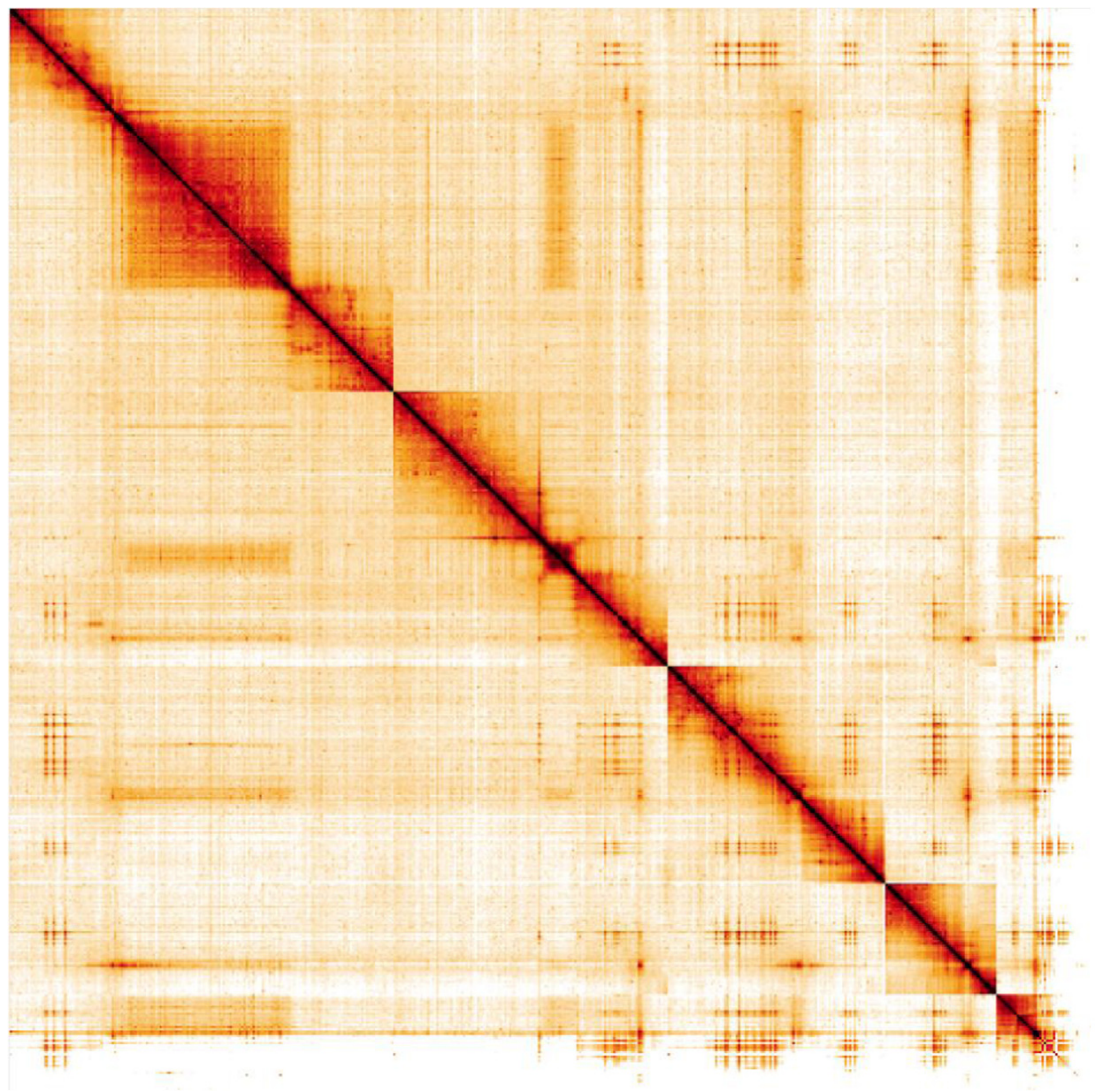
Genome assembly of *Xanthogramma pedissequum*, idXanPedi1.1: Hi-C contact map. Hi-C contact map of the idXanPedi1.1 assembly, visualised in HiGlass. Chromosomes are given in order of size, from left to right and top to bottom.

**Table 2.** Chromosomal pseudomolecules in the genome assembly of *Xanthogramma pedissequum*, idXanPedi1.1.

INSDC accession	Chromosome	Size (Mb)	GC%
OU343160.1	1	346.88	34.5
OU343161.1	2	248.69	33.8
OU343162.1	3	195.21	33.2
OU343163.1	4	101.21	33.3
OU343164.1	X	38.39	34.5
OU343165.1	Y	2.99	36.1
OU343166.1	MT	0.02	16.8
-	Unplaced	43.79	36.0

## Methods

### Sample acquisition and nucleic acid extraction

A single male Xanthogramma pedissequum was collected from Wigmore Park (51.88378, -0.36861422), Percival Way, Wigmore, Luton, England, by Olga Sivell, Natural History Museum, London, using a sweep net. The morphological identification was provided by Duncan Sivell, Natural History Museum, London, based on Stubbs, Falk, and Others 2002; Ball *et al.,* 2015; Speight, 2010; Speight & Sarthou, 2017; Stubbs, 2012a). The sample was snap-frozen using dry ice and stored in a CoolRack.

DNA was extracted at the Tree of Life laboratory, Wellcome Sanger Institute. The idCorMarg1 sample was weighed and dissected on dry ice with tissue set aside for Hi-C sequencing. Thorax tissue was disrupted to a fine powder using a Nippi Powermasher fitted with a BioMasher pestle. Fragment size analysis of 0.01–0.5 ng of DNA was then performed using an Agilent FemtoPulse. High molecular weight (HMW) DNA was extracted using the Qiagen MagAttract HMW DNA extraction kit. Low molecular weight DNA was removed from a 200-ng aliquot of extracted DNA using 0.8X AMpure XP purification kit prior to 10X Chromium sequencing; a minimum of 50 ng DNA was submitted for 10X sequencing. HMW DNA was sheared into an average fragment size between 12–20 kb in a Megaruptor 3 system with speed setting 30. Sheared DNA was purified by solid-phase reversible immobilisation using AMPure PB beads with a 1.8X ratio of beads to sample to remove the shorter fragments and concentrate the DNA sample. The concentration of the sheared and purified DNA was assessed using a ThermoFisher Nanodrop spectrophotometer and Qubit Fluorometer and Qubit dsDNA High Sensitivity Assay kit. Fragment size distribution was evaluated by running the sample on the FemtoPulse system.

### Sequencing

Pacific Biosciences HiFi circular consensus and 10X Genomics read cloud DNA sequencing libraries were constructed according to the manufacturers’ instructions. DNA sequencing was performed by the Scientific Operations core at the WSI on Pacific Biosciences SEQUEL II (HiFi) and Illumina HiSeq X (10X) instruments. Hi-C data were generated from further thorax tissue from the same specimen using the Arima Hi-C+ kit and sequenced on an Illumina NovaSeq 6000 instrument.

### Genome assembly

Assembly was carried out with Hifiasm ( Cheng *et al*., 2021); haplotypic duplication was identified and removed with purge_dups ( Guan *et al*., 2020). One round of polishing was performed by aligning 10X Genomics read data to the assembly with longranger align, calling variants with freebayes ( Garrison & Marth, 2012). The assembly was then scaffolded with Hi-C data ( Rao *et al*., 2014) using SALSA2 ( Ghurye *et al*., 2019). The assembly was checked for contamination as described previously ( Howe *et al*., 2021). Manual curation ( Howe *et al*., 2021) was performed using HiGlass ( Kerpedjiev *et al*., 2018) and Pretext. The mitochondrial genome was assembled using MitoHiFi ( Uliano-Silva *et al*., 2021), which performed annotation using MitoFinder ( Allio *et al*., 2020). The genome was analysed and BUSCO scores generated within the BlobToolKit environment ( Challis *et al*., 2020). Table 3 contains a list of all software tool versions used, where appropriate.

**Table 3.** Software tools used.

Software tool	Version	Source
Hifiasm	0.12	Cheng *et al*., 2021
purge_dups	1.2.3	Guan *et al*., 2020
SALSA2	2.2	Ghurye *et al*., 2019
longranger align	2.2.2	https://support.10xgenomics.com/genome- exome/software/pipelines/latest/advanced/other- pipelines
freebayes	1.3.1-17-gaa2ace8	Garrison & Marth, 2012
MitoHiFi	2.0	Uliano-Silva *et al*., 2021
HiGlass	1.11.6	Kerpedjiev *et al*., 2018
PretextView	0.2.x	https://github.com/wtsi-hpag/PretextView
BlobToolKit	2.6.4	Challis *et al*., 2020

### Ethics/compliance issues

The materials that have contributed to this genome note have been supplied by a Darwin Tree of Life Partner. The submission of materials by a Darwin Tree of Life Partner is subject to the Darwin Tree of Life Project Sampling Code of Practice. By agreeing with and signing up to the Sampling Code of Practice, the Darwin Tree of Life Partner agrees they will meet the legal and ethical requirements and standards set out within this document in respect of all samples acquired for, and supplied to, the Darwin Tree of Life Project. Each transfer of samples is further undertaken according to a Research Collaboration Agreement or Material Transfer Agreement entered into by the Darwin Tree of Life Partner, Genome Research Limited (operating as the Wellcome Sanger Institute), and in some circumstances other Darwin Tree of Life collaborators.

## Data availability

European Nucleotide Archive: *Xanthogramma pedissequum* (hoverfly). Accession number PRJEB45174; https://identifiers.org/ena.embl/PRJEB45174.

The genome sequence is released openly for reuse. The *X. pedissequum* genome sequencing initiative is part of the Darwin Tree of Life (DToL) project. All raw sequence data and the assembly have been deposited in INSDC databases. The genome will be annotated and presented through the Ensembl pipeline at the European Bioinformatics Institute. Raw data and assembly accession identifiers are reported in Table 1.

## Author information

Members of the Natural History Museum Genome Acquisition Lab are listed here: https://doi.org/10.5281/zenodo.5746819.

Members of the Darwin Tree of Life Barcoding collective are listed here: https://doi.org/10.5281/zenodo.5744972.

Members of the Wellcome Sanger Institute Tree of Life programme are listed here: https://doi.org/10.5281/zenodo.5744840.

Members of Wellcome Sanger Institute Scientific Operations: DNA Pipelines collective are listed here: https://doi.org/10.5281/zenodo.5746904.

Members of the Tree of Life Core Informatics collective are listed here: https://doi.org/10.5281/zenodo.5743293.

Members of the Darwin Tree of Life Consortium are listed here: https://doi.org/10.5281/zenodo.5638618.
