# Supplementary material for: The genome sequence of a hoverfly, Xanthogramma pedissequum (Harris, 1776)
Source: Wellcome Open Res. 2022 Feb 2;7:38. [Version 1] doi: 10.12688/wellcomeopenres.17559.1 (PMC8831851; doi:10.12688/wellcomeopenres.17559.1)
Supplement: Peer review history

# Peer review: Reviewer response for version 1

**DOI:** 10.21956/wellcomeopenres.19416.r48478

**Published:** 2022-02-10

**Authors:** Ching-Ho Chang

**Affiliations:** 1 Division of Basic Sciences, Fred Hutchinson Cancer Research Center, Seattle, WA, 98109, USA

Sivell *et al.* report a genome assembly of a hoverfly, *Xanthogramma pedissequum*. The high completeness and contiguity indicate that genome assembly quality is very high. However, I feel that some extra analyses would greatly improve the significance of the study. I also found that the description of methods can be more detailed.

 My major suggestion is about the chromosome evolution of the Dipteran lineage. Dipteran species have been known to have a large gene synteny, which is named Muller element, as it was first described by H. J. Muller (Muller 1940 ^1^). Muller labeled *Drosophila* chromosome arms as A to F and found genes located in one Muller element seldom translocated to other Muller elements in other species. Recent studies found Muller elements are also conserved in other Dipteran species, e.g., mosquitos. The nomenclature is particularly useful for people to study chromosome evolution. Can the author also assign hoverfly’s chromosomes using Muller elements?

 The other major suggestion is that sex chromosomes have turned over several times in Dipterans (Vicoso and Bachtrog, 2015 ^1^). Can the authors state the evolution of sex chromosomes in *Xanthogramma pedissequum* based on their observation?

 I also have a minor question. There is no bacteria or fungus contamination in their assembly. Is that true, or did the authors use some ways to filter out their contaminating contigs?

Are sufficient details of methods and materials provided to allow replication by others?

Yes

Is the rationale for creating the dataset(s) clearly described?

Yes

Are the datasets clearly presented in a useable and accessible format?

Yes

Are the protocols appropriate and is the work technically sound?

Yes

Reviewer Expertise:

Genomics, Genetics

I confirm that I have read this submission and believe that I have an appropriate level of expertise to confirm that it is of an acceptable scientific standard.

## References

[1] : Bearings of the 'Drosophila’ work on systematics. *J. Huxley (ed.), The New Systematics* .1940;185-268

[2] : Numerous transitions of sex chromosomes in Diptera. *PLoS Biol* .2015;13(4) : 10.1371/journal.pbio.1002078 e1002078 10.1371/journal.pbio.1002078 25879221PMC4400102

# Peer review: Reviewer response for version 1

**DOI:** 10.21956/wellcomeopenres.19416.r48481

**Published:** 2022-02-08

**Authors:** Emma Bailey

**Affiliations:** 1 Department of Biointeractions and Crop Protection, Rothamsted Research, Harpenden, UK

The Darwin Tree of Life project report a high quality genome assembly for a Syrphidae hoverfly ( *Xanthogramma pedissequum*). This has been performed using PacBio and 10X sequencing data, with scaffolding using Hi-C data. All raw data and the genome assembly are easily accessible. This will make a useful contribution to further research of hoverflies, many of which are key pollinators and beneficial predators of crop pests.

**Background**

 This gives a good overview of the biology of *X. pedissequum, *highlighting their potentially important role as aphid predators. 

**Genome sequence report**

 "The final assembly has a total length of 977 Mb" - Was any genome size estimate obtained for this species to reaffirm this value, using flow cytometry or K-mer analysis? 

 "The majority, 95.94%, of the assembly sequence was assigned to six chromosomal-level scaffolds" - Can any comment be made on whether this was the expected number of chromosomes based on closely related species?

 Statistics on the repeat content of the genome would be useful, especially considering the "high repeat content" reported in chromosome 1.  But perhaps this will follow with subsequent annotation.

 I don't personally feel that figures 3 and 4 add much to the report. However, as they appear to be included as a standard for all DToL genome reports, I see no harm in their inclusion. 

 Figure 5 - it is difficult to identify individual chromosomes on the contact map, is it possible to outline these? 

**Methods**

 Wet lab methods are covered well. Genome assembly methods are brief, no parameters are mentioned, so I'm assuming settings were all kept as default? It might be worth mentioning this if so.

 Additionally, it could be useful to include a table of assembly statistics before and after purge_dups, polishing and scaffolding, but this is not essential. 

Are sufficient details of methods and materials provided to allow replication by others?

Partly

Is the rationale for creating the dataset(s) clearly described?

Yes

Are the datasets clearly presented in a useable and accessible format?

Yes

Are the protocols appropriate and is the work technically sound?

Yes

Reviewer Expertise:

Insect genomics/bioinformatics

I confirm that I have read this submission and believe that I have an appropriate level of expertise to confirm that it is of an acceptable scientific standard.
